# Peculiarities of the Transformation of *Asteraceae* Family Species: The Cases of Sunflower and Lettuce

**DOI:** 10.3389/fpls.2021.767459

**Published:** 2021-11-26

**Authors:** Flavia Soledad Darqui, Laura Mabel Radonic, Valeria Cecilia Beracochea, H. Esteban Hopp, Marisa López Bilbao

**Affiliations:** ^1^IABIMO (Instituto de Agrobiotecnología y Biología Molecular), UEDD INTA-CONICET, CNIA, Buenos Aires, Argentina; ^2^Departamento de Fisiología, Biología Molecular y Celular (FBMC), Facultad de Ciencias Exactas y Naturales (FCEyN), Universidad de Buenos Aires (UBA), Ciudad Autónoma de Buenos Aires, Buenos Aires, Argentina

**Keywords:** transgenesis, sunflower, lettuce, model species, *Asteraceae*

## Abstract

The *Asteraceae* family is the largest and most diversified family of the Angiosperms, characterized by the presence of numerous clustered inflorescences, which have the appearance of a single compound flower. It is estimated that this family represents around 10% of all flowered species, with a great biodiversity, covering all environments on the planet, except Antarctica. Also, it includes economically important crops, such as lettuce, sunflower, and chrysanthemum; wild flowers; herbs, and several species that produce molecules with pharmacological properties. Nevertheless, the biotechnological improvement of this family is limited to a few species and their genetic transformation was achieved later than in other plant families. Lettuce (*Lactuca sativa* L.) is a model species in molecular biology and plant biotechnology that has easily adapted to tissue culture, with efficient shoot regeneration from different tissues, organs, cells, and protoplasts. Due to this plasticity, it was possible to obtain transgenic plants tolerant to biotic or abiotic stresses as well as for the production of commercially interesting molecules (molecular farming). These advances, together with the complete sequencing of lettuce genome allowed the rapid adoption of gene editing using the CRISPR system. On the other hand, sunflower (*Helianthus annuus* L.) is a species that for years was considered recalcitrant to *in vitro* culture. Although this difficulty was overcome and some publications were made on sunflower genetic transformation, until now there is no transgenic variety commercialized or authorized for cultivation. In this article, we review similarities (such as avoiding the utilization of the *CaMV35S* promoter in transformation vectors) and differences (such as transformation efficiency) in the state of the art of genetic transformation techniques performed in these two species.

## Introduction

*Asteraceae* is the largest and most diversified Angiosperm family (Funk et al., 2005). With more than 24,000 described species, it is estimated that this family represents about 10% of all flowering species. It includes economically important crops, wild flowers, herbs, and several species that contain molecules of medical interest (Dempewolf et al., 2008). Domesticated crops include food crops (lettuce, chicory, and topinambur), oil (sunflower and safflower), medicinal (*Echinacea* and chamomile), and many ornamentals (chrysanthemum, dahlia, zinnia, gerbera, and others). Alternatively known as *Compositae*, this family is characterized by the presence of numerous grouped inflorescences that have the appearance of a single “compound” flower. It is divided into three major subfamilies and a minor subfamily, with lettuce, sunflower, and safflower being the agronomically important representatives of the major subfamilies. They present a great biodiversity encompassing the extreme environments on the planet and not only in the areas between the tropics, as occurs in the rest of the Angiosperms. With the exception of Antarctica, representatives of this family are found in all environments and continents (Funk et al., 2005).

The study and application of biotechnological techniques related to *in vitro* culture and transformation in species of the *Asteraceae* family have been developed mainly in chrysanthemum, lettuce, and sunflower. In the case of chrysanthemum, one of the most important cut flowers and ornamental plants used all over the world, important advances were made on different biotechnological aspects detailed in numerous research and reviewed by Darqui et al. (2017) and Boutigny et al. (2020). Unlike other ornamental species, *in vitro* micropropagation *via* somatic embryos or shoot regeneration is not used for chrysanthemum large-scale production. There are no reports of varieties generated from somatic hybrids, cryopreservation is not used to maintain existing varieties and unlike other ornamental plants, such as carnation or rose, there are no transgenic chrysanthemum varieties available on the market (Visser et al., 2007; Chandler and Sanchez, 2012) even though an authentic blue chrysanthemum was obtained (Noda et al., 2017).

Sunflower is one of the most important sources of edible oil and total world sunflower seed production, 52million tons for 27million ha in 2018, goes almost exclusively to oil extraction, providing 9% of total world volume (Pilorgé, 2020). Its oil is considered of good quality because of its light taste and appearance but especially because it supplies more vitamin E than any other vegetable oil. Sunflower and peanut are the only major vegetable oil-yielding crops that have no genetically modified (GM) varieties authorized for commercial use. Sunflower biotechnological improvement is limited to molecular marker-assisted selection and transgenic sunflower can only be found in controlled and experimental environments.

Lettuce, on the other hand, is a model species in cell biology and biotechnology research, due among other factors, to its good response to tissue culture. Lettuce is a leafy vegetable that is globally grown and widely consumed (Kim et al., 2016). The development of a stable transformation system in lettuce has enabled the introduction of many potentially useful genes in this crop, oriented to the molecular breeding of lettuce itself as well as to the production of molecules of economic interest. This model plant has also been selected as a platform for recombinant production of miraculin, a taste-modifying glycoprotein extracted from the red berries of the West African native shrub *Richadella dulcifica* (Hirai et al., 2011). Lettuce has advantages for biotechnology applications, for instance, it can be eaten fresh allowing the preservation of proteins. Its adaptability to greenhouse conditions and hydroponic culture allows cultivation, that can be easily scaled up or down, in controlled environments. As a plant bioreactor, its life cycle is shorter than in other plant alternatives allowing the recovery of the product of interest in a short term.

Although these three vegetable species are very different in many aspects, they may present unwanted responses when the *CaMV35S* promoter, the most used constitutive promoter in plant biotechnology, is used for plant transformation. During the mid-1990s, it became increasingly clear that this promoter was less active in chrysanthemum than in, for example, tobacco (Outchkourov et al., 2003). In lettuce, the expression level was as high as in other species but the transgene was unstable and lost after two or three generations (Davey et al., 2007), although still used in most studies. In sunflower, vectors containing *CaMV35S* promoter regulating *β-glucuronidase* gene (GUS) showed a low and non-constitutive expression pattern in T1 plants while the detection of transgenes was not possible in T2 plants due to its genetic instability (Radonic, 2010).

The development of efficient *Asteraceae* transformation systems has often been combined with the transfer and assessment of target genes for traits, such as plant architecture and resistance to biotic and abiotic stresses. The use of suboptimal transformation protocols and expression vectors resulted in a considerable number of reports with some independent lines with low expression of the transgene, so there is unclear or doubtful data about inserted genes effects. This situation is evident in the transformation of sunflower, while in the case of lettuce, it could be partially overcome due to its good response and adaptation to *in vitro* cultivation. In this review, we focus on these two species with such contrasting behavior in response to plant *in vitro* culture and transformation efficiency.

## Sunflower

There is a worldwide interest of growers in sunflower improvement, demonstrated by the rapid adoption of the first herbicide-tolerant non-GM sunflower (Clearfield^®^). This trait consists of an imidazolinone (IMI) genetic resistance identified in a wild population of *H. annuus* (Miller and Al-Khatib, 2002) that was incorporated into elite germplasm through conventional breeding. However, no transgenic sunflower has reached cultivation and commercialization approval in any country so far. This shows the existing difficulties in sunflower breeding by transgenesis.

Since the first studies referring to sunflower *in vitro* culture, difficulties were evident in the regeneration step even from diverse explant sources and with different systems. The first publications discarded shoots or embryo regeneration from callus (Greco et al., 1984; Paterson and Everett, 1985; Wilcox McCann et al., 1988). Besides, *in vitro* culture response is highly genotype dependent (Fiore et al., 1997; Gürel and Kazan, 1998). The few advances obtained in tissue culture from cotyledons, immature embryos, shoot tips, protoplasts, hypocotyls, or leaves are detailed in Moschen et al. (2014) or in Dagustu (2018) where the more than 50 reviewed articles show that sunflower is a still difficult species to deal with because of its low regeneration ability. Different research groups continue working in solving this issue and trying to improve sunflower *in vitro* culture. For example, Zhang and Finer (2016b) described the use of a cytokinin pulse treatment for shoot induction followed by the use of gibberellic acid (GA) for shoot development and elongation, combining with micrografting for high-efficiency recovery of plants from developed shoots. Similarly, Islam et al. (2021) have performed *in vitro* direct organogenesis from cultured seed of sunflower. Aurori et al. (2020) tested various auxin treatments trying to analyze the morphogenetic potential of a new explant, the apex with primordial leaves resulting from ungerminated mature zygotic embryos.

The poor response in tissue culture directly affected the development and obtaining of transgenic sunflower. This is due to the necessity of the transformation of as many cells as possible and with a good regeneration potential to ensure the success in genetic transformation. To summarize, most published transformation systems for sunflower are based on the work of Alibert et al. (1999), explained in detail in Radonic et al. (2015), consisting of the following steps: imbibition of seeds, excision of embryonic axes, co-culture with *Agrobacterium tumefaciens*, induction of shoots, and recovery of transformed shoots, selection, shoot elongation, transfer to greenhouse, and acclimatization. This scheme produces, by direct organogenesis, one or two shoots per explant derived from the association of several cells of each explant, so they are frequently chimeric (Schrammeijer et al., 1990) where transgenic sectors may not lead to the recovery of transgenic progeny (Burrus et al., 1996). A recently review on sunflower transgenesis (Sheri et al., 2020) lists 51 publications describing the transformation method used (mostly using *Agrobacterium*, but also gene gun or a combination of both methods), the genes introduced and a brief description of the characteristics obtained.

In order to overcome the low levels of transformation due to the poor response to tissue culture and low regeneration rate, Molinier et al. (2002) used the *ipt* gene to induce transient expression of cytokinins. The latest attempt to improve *in vitro* regeneration was the overexpression of the developmental transcription factor *growth-regulating factor 5* (*GRF5*) gene from *Arabidopsis thaliana* or its orthologous (Kong et al., 2020). This strategy significantly increased the frequency of shoots expressing green fluorescent protein (GFP) marker protein per explant. Nevertheless, transformation efficiency was not always enhanced, suggesting that this increase in transgenic shoot number is due to the proliferation of more shoots from the same transformed explants. This research opens an interesting opportunity to adjust the use of *GRF5*-induced regeneration and to improve the transgenesis protocol.

Regarding selection genes, *neomycin phosphotransferase* (*nptII*) gene is still the most widely used. However, the only reliable characteristic for the screening of transgenic shoots was the *in vitro* formation of roots and not the bleaching green tissues in presence of kanamycin (Radonic et al., 2006). There are only a few cases where other selection agents were used, such as the hygromycin antibiotic (*hpt* gene; Benzle et al., 2015; Zhang and Finer, 2016a) and the phosphinothricin herbicide, in its variants glufosinate, BASTA^®^, or bialaphos (*bar* gene; Escandon and Hahne, 1991; Neskorodov et al., 2010).

Zhang and Finer (2016b) developed another approach to improve sunflower transformation efficiency using a low inoculum of *Agrobacterium* at about 6×10^2^CFUml^−1^ with a long co-culture period of 15days, which avoid the suppression of plant regeneration or activation of defense responses. This relatively low amounts of bacteria mimics the natural infestation of plants by *Agrobacterium*, which led to an average of three transformed shoots per explant (in a 20% of treated explants) while the use of a typical co-culture method did not produce transformed shoots. Nevertheless, this study was restricted to T0 plants and there is no information about the transgene stability or expression level obtained. A further analysis of this strategy must be considered.

Besides the previously mentioned difficulties in sunflower transformation, what it appears to be one of the causes to prevent the success in obtaining sunflower transgenic lines is the use of the constitutive *CaMV35S* promoter (Radonic, 2010). *CaMV35S* promoter (Benfey and Chua, 1990) is the most widely used sequence for gene expression during the genetic transformation of plant species, both monocotyledonous and dicotyledonous, regulating the expression of reporter, selection, or interest genes and it is present in different highly popular vectors like pCAMBIA, pPZP, pGWB, Gateway. However, despite the success in the use of this promoter, some studies described expression patterns different from that expected for a constitutive promoter. In the case of the *Asteraceae* family, the lack of β-glucuronidase activity under the regulation of this promoter was described in chrysanthemum (Annadana et al., 2001; Outchkourov et al., 2003). These difficulties led to the use of new alternative promoters to express genes in both leaf and flower (Annadana et al., 2002a,b; Outchkourov et al., 2003; Aida et al., 2004, 2005).

In *A. thaliana*, Yoo et al. (2005) described an effect in *trans* of the *CaMV35S* promoter, which affected and altered the expression pattern of tissue-specific transgenes, modifying the phenotype of transgenic plants. This interference disappeared when this promoter was replaced, suggesting that these effects were caused by the enhancer of the *CaMV35S* promoter. Subsequently, Zheng et al. (2007) described that the presence of the *CaMV35S* promoter transformed specific promoters *AGL5* (ovarian-specific), *PAB5* (early embryogenesis-specific), and *AAP2* (reproductive and vascular tissue-specific) into constitutive ones. Also in arabidopsis, Gudynaite-Savitch et al. (2009) studied the interference of *CaMV35S* promoter regulating the selection gene, over specific promoters which lose their transcriptional specificity. This effect disappeared when the *CaMV35S* promoter was replaced or the distance among promoters was increased in the vector. Additionally, they found that this negative interaction was promoter dependent suggesting that it should be studied for each promoter combination.

Transgenic sunflower events expressing the *β-glucuronidase* reporter gene under the *CaMV35S* promoter showed low expression levels, even when using different selection genes (*bar* or *nptII*) in the same transformation vector (Radonic et al., 2006). In all cases, GUS staining was detected in a specific-tissue pattern in the trichomes of young leaves, being necessary the use of stereoscopic magnifying glasses. Additionally, the instability of the transgenes in T1 events was observed.

**Figure 1:**
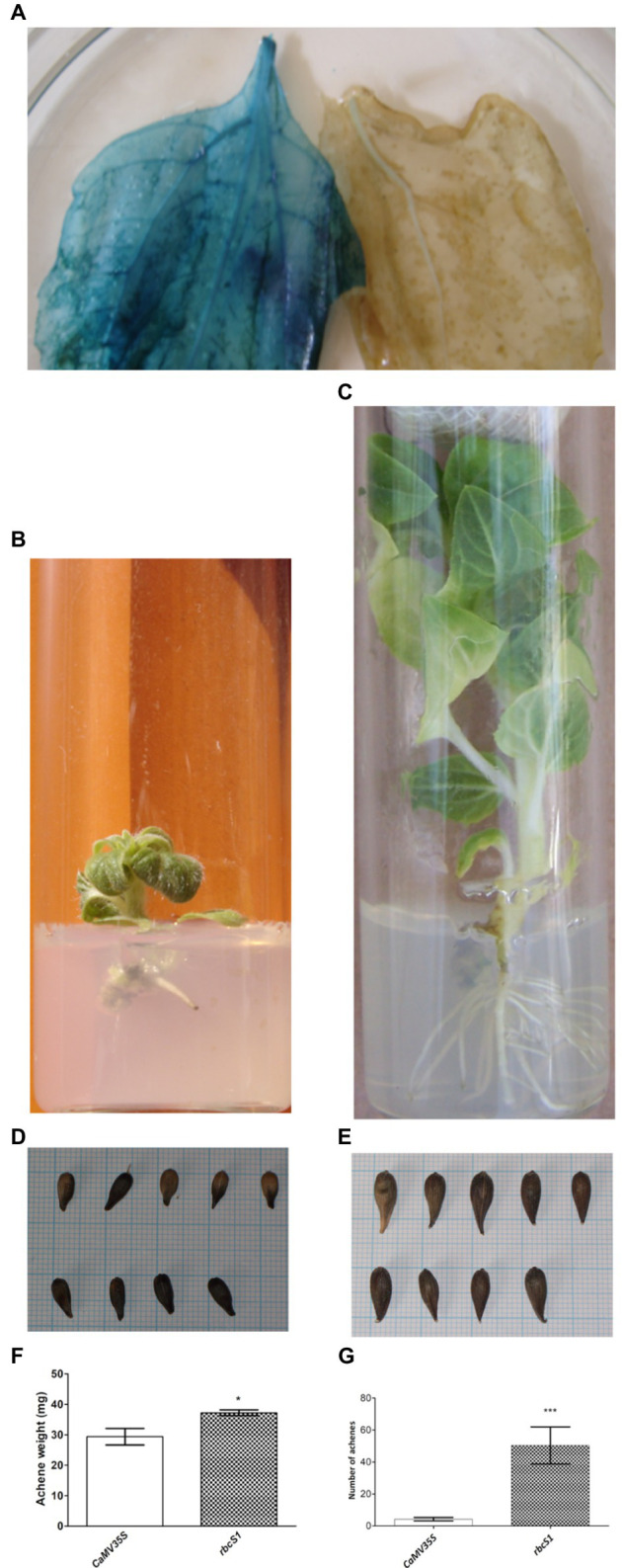
Comparison of transgenic sunflowers carrying constructions with either *CaMV35S* or *rbcS1* promoters. GUS histochemical staining of leaves from *rbcS1* promoter-*GUS* gene transgenic (left) and control (right) sunflower plants **(A)**. *In vitro* root development in kanamycin with constructions carrying either *CaMV35S* **(B)** or *rbcS1* **(C)** promoters. Sizes of achenes from transgenic sunflowers carrying constructions with either *CaMV35S* **(D)** or *rbcS1* **(E)** promoters. Weight of achenes in mg ^*^p<0.01 (*t*-test) **(F)** and number of achenes per floral chapter ^***^p<0.001 (Welch’s *t*-test) **(G)** from transgenic sunflowers carrying constructions with either *CaMV35S* or *rbcS1* promoters. Source: Radonic (2010).

The replacement of *CaMV35S* promoter by the chrysanthemum *rbcS1* (*ribulose bisphosphate carboxylase small subunit*) promoter improved transformation efficiency in sunflower, increasing the number of seedlings capable of rooting in kanamycin and, for the first time, a typical constitutive expression of the *GUS* reporter gene (Figure 1A) was observed in T1 plants (Radonic, 2010). This suggested that *CaMV35S* promoter was affecting in *trans* the expression of the selection gene regulated by the *nos* promoter, as mentioned in the preceding paragraphs. In addition, the seedlings obtained with the *rbcS1* promoter presented a healthy appearance and most of them were able to develop abundant roots in selective medium (Figures 1B,C). Also, these shoots were successfully transferred to the greenhouse, producing bigger plants with larger flower heads and a significant increase in the number and size of achenes (Figures 1D–G).

Most studies in sunflower transformation use *CaMV35S* or *2XCaMV35S* promoter to direct the expression of the gene of interest or the marker gene, and even recently, *2XCaMV35S* promoter was used to obtain plants with improved salinity tolerance (Mushke et al., 2019). There are some exceptions: The petunia *FBP1* promoter used to control the *HAM59 MAD*-box gene (Shulga et al., 2015), and *scp1* and *supermas* promoters overexpressing the wheat *OXO* gene (Hu et al., 2003). About this last research, it must be highlighted that homozygous T4 plants were evaluated. This is remarkable because most sunflower transformation studies show results obtained in T0 and T1 generations. This could be due to difficulties in obtaining plants in successive progeny (T2 or further), possibly because of transgene instability caused by the use of the *CaMV35S* promoter. Information provided by T0 is very limited as the obtained plants are generally chimeras; then, it is possible that inflorescences are not transformed, producing non-GM descendants.

The only study that showed a stabilized T4 sunflower transgenic line carrying the *CaMV35S* promoter was Singareddy et al. (2018). They reached this generation with five events, although they transformed 24,328 explants. These authors experienced the problems mentioned in the previous paragraphs as they described that only 20 plants that reached maturity in T0 produced seeds that germinated successfully, while other plants produced one or two seeds either filled or vain.

## Lettuce

Contrary to sunflower, lettuce is very amenable to different *in vitro* culture techniques (Doerschug and Miller, 1967; Michelmore and Eash, 1986). Consequently, effective lettuce transformation protocols were early established (Michelmore et al., 1987) by both callus induction and shoot regeneration using different genotypes showing their tissue culture responsiveness (Curtis et al., 1994). Since then, many articles were published on lettuce transgenesis, many of which were revised in previous publications (Davey et al., 2007; Song et al., 2014). Thus, this work will focus on technical progress achieved on lettuce transformation during the last decade.

In addition to its responsiveness to tissue culture and genetic transformation, lettuce has characteristics that makes it a model species: it has a short life cycle, which allows a rapid recovery of complete plants, molecules of interest, and transgenic progeny, it is an autogamous plant with a completely sequenced diploid genome (Reyes-Chin-Wo et al., 2017), which facilitates the obtaining of homozygous transgenic lines, and it can be cultured and grown at chamber, in hydroponics, in the greenhouse or in the field, allowing an easy scaling-up or -down of its production. As a model species, lettuce has been widely used for the characterization of endogenous genes, like *9-cis-epoxycarotenoid dioxygenase 4* (*NCED4*; Huo et al., 2013), small rubber particle protein genes *LsSRPP4* and *LsSRPP8* (Chakrabarty et al., 2014) and *cisprenyltransferase-like 2* (*CPTL2*; Qu et al., 2015), or foreign genes, such as heat shock protein gene *AtHSP17.8* (Kim et al., 2013) and glycine-rich protein gene *AtGRDP2* (Ortega-Amaro et al., 2015) from arabidopsis or the C3HC4-type RING zinc finger gene *CaRZFP1* from pepper (Kesawat et al., 2018). Lettuce is a particularly interesting model system in functional genomics studies within the *Asteraceae* family (Moschen et al., 2014), as it is phylogenetically related with these species. For example, lettuce has been used to study the tissue specificity of the sunflower *HaAP10* promoter, which directed GUS expression in lettuce seeds (Zavallo, 2011).

Furthermore, lettuce is one of the most used species in molecular farming strategies, because it grows quickly under greenhouse conditions and can produce a high amount of green biomass that can be consumed fresh or lyophilized. This make lettuce tissues promising vehicles for biopharmaceutical production, for example, in the production of plant-based oral vaccines, which are considered alternatives or supplements to standard injection vaccines, with the possibility to simplify vaccination procedures. One example is the obtaining of lettuce plants expressing a small surface antigen of *hepatitis B virus* (S-HBsAg), which progressed toward the use of lettuce tissues for oral vaccination in mice (Pniewski et al., 2011), or the development of lettuce plants expressing a double Shiga toxin 2e (Stx2e) from enterohemorrhagic *Escherichia coli* and the oral vaccination of piglets to induce protection against pig edema disease (Matsui et al., 2011; Hamabata et al., 2019).

Moreover, lettuce has the potential to ameliorate genetic or infectious diseases through RNA interference (RNAi) approaches. Its endogenous molecular machinery was exploited to express artificial microRNAs (amiRNAs) against HBsAg and the administration of a lettuce decoction in HBsAg−/+ transgenic mice inhibited the expression of this gene (Zhang et al., 2019). Lettuce was also used to produce amiRNAs against complement 3 (C3) and coagulation factor 7 (CF7) mouse mRNAs, two proteins whose excessive production can cause blood clots (Kakeshpour et al., 2019).

A specialty of molecular farming is the use of transplastomic technology, which facilitates the expression of molecules of interest, by expressing 10,000 copies of transgene per cell. It also has the advantage that plastid DNA is absent in pollen, avoiding foreign gene escapes into the environment. Successful examples of lettuce transplastomic platforms are the production of therapeutic human proteins Thioredoxin 1 (Lim et al., 2011) and Granulocyte Colony-Stimulating factor (Tabar et al., 2013) or the production of a Dengue virus tetra-epitope peptide (Maldaner et al., 2013). Also, there is a study showing efficient expression of coagulation factor IX for hemophilia treatment (Su et al., 2015). Another example is the expression of the poliovirus capsid protein (VP1) fused with the *Cholera* non-toxic B subunit (CTB) in lettuce chloroplasts for the development of a virus-free oral polio booster vaccine (Daniell et al., 2019) or the production of codon optimized Pro-insulin-like growth factor-1 (Pro-IGF-1) fused with the CTB transmucosal carrier or cell penetrating transduction domain (PTD), for treatment of musculoskeletal diseases (Park et al., 2020).

However, there are cases in which transplastomic lettuce plants were unable to produce the desired protein. For example, expression of an immunogenic protein against the influenza virus chloroplast was attempted without success, although northern blot analyses demonstrated transgene expression, the heterologous protein was not identified (Lelivelt et al., 2005). Another example is the *rhBMP2* gene, which encodes a human protein participating in bone and cartilage regeneration, that was introduced in two regions of the lettuce chloroplast genome, but although the presence of steady-state mRNA was confirmed, it was not possible to detect BMP2 proteins in leaves (Queiroz et al., 2019). However, BMP was reported to be a difficult-to-produce protein in several expression systems including *E. coli*, *Pichia pastoris*, insect, and mammalian cells because of the impairment of protein folding and post-translational modifications (Ceresoli et al., 2016).

The development of a stable transformation system in lettuce allowed the introduction of many genes toward crop breeding. Recent studies on biotic stress-resistance improvement include transgenic plants expressing antimicrobial peptides, like rabbit defensin NP-1, which demonstrated antimicrobial activity against *Bacillus subtilis* and *Pseudomonas aeruginosa* in *in vitro* assays using transgenic extracts (Song et al., 2017) or potato *snakin-1* overexpressing plants which showed *in vivo* enhanced tolerance against necrotrophic fungi *Sclerotinia sclerotiorum* and *Rhizoctonia solani* (Darqui et al., 2018b).

The RNAi-based technology was also used to obtain *mirafiori lettuce big-vein virus* (MLBVV) resistant plants by the introduction of inverted repeats of the coat protein gene (Kawazu et al., 2016), plants expressing siRNAs that suppressed Highly Abundant Message #34 (HAM34) or Cellulose Synthase (CES1) *Bremia lactucae* genes, thus reducing *B. lactucae* growth and sporulation (Govindarajulu et al., 2015) or plants expressing dsRNAs targeting whitefly v-ATPase transcripts, to interfere with the insect life cycle (Ibrahim et al., 2017).

In relation to abiotic stress-resistance, the overexpression of arabidopsis heat shock protein AtHSP17.8 led to hypersensitivity to ABA and enhanced lettuce resistance against dehydration and high salinity stresses (Kim et al., 2013).

In recent years, advances were obtained in different aspects of lettuce transformation protocols. For example, improvements were made to optimize cell suspension culture conditions, including hormonal combinations, pH, temperature, and salt concentrations, of *rol ABC*- and *rol C*-transformed lines, in order to increase biomass for a large-scale production of secondary metabolites (Ismail et al., 2019).

Regarding selection methods, 75mg/L of kanamycin was the optimum threshold concentration to select kanamycin-resistant transgenic plantlets while avoiding escapes, when using the selection cassette p*nos*-*nptII*-t*nos*. Morphological responses of transgenic and non-transgenic seedlings to kanamycin were evaluated and lateral root development showed an early, qualitative and reliable association with *nptII* presence, as corroborated by PCR detection. This method allowed a simplified scaling-up of the production of multiple homozygous transgenic progeny lines in early generations, avoiding expensive and time-consuming molecular assays (Darqui et al., 2018a).

Also, a lettuce micropropagation protocol using axillary buds was developed to avoid the risk of somaclonal variation during the mass-scale production of transgenic lines carrying HBV surface antigens (Pniewski et al., 2017).

In addition, the relative ease achieved in lettuce transformation facilitated a rapid adoption of CRISPR-mediated gene editing. This is reflected in the rapid development of gene editing protocols by protoplasts transfection (Woo et al., 2015; Park et al., 2019) or by *A. tumefaciens*-mediated transformation (Bertier et al., 2018; Zhang et al., 2018; Luo et al., 2020; Yu et al., 2020). In most of these studies, development and/or hormonal regulation-related genes were knocked-out.

Although lettuce is amenable to plant transformation and tissue regeneration, the accomplishment in obtaining new varieties will depend not only on the effectiveness of the transformation protocols but also on the selected gene construct. Even though it seemed to be relatively easy to introduce foreign DNA into lettuce germplasm, transgenes are not always correctly expressed or inherited.

For example, as in sunflower, the *CaMV35S* promoter was the most traditionally used promoter for expression of genes in lettuce transformation. However, difficulties in the use of this genetic element were also detected. McCabe et al. (1999) observed that 97% of T0 plants transformed with the *bar* gene under the plastocyanin promoter from pea (*pet*E)-transmitted herbicide resistance until the T3 generation, while only 2.5% of the T0 plants transformed with *CaMV35S-bar* transferred this phenotype to the third generation. In addition, miraculin expression under the ubiquitin promoter was higher and more stable than under the *CaMV35S* promoter (Hirai et al., 2011). Moreover, site-specific promoter methylation resulted in transgene silencing in *CaMV35S-GFP* plants (Okumura et al., 2015).

In view of this background reports, it was decided to use the *rbcS1* promoter to drive the expression of the antimicrobial peptide Snakin-1 (Darqui et al., 2018b). This plant promoter from an *Asteraceae* species, which has shown to increase expression levels compared to the *CaMV35S* promoter in sunflower (Radonic, 2010), directed a stable expression in lettuce of *snakin-1* in T4 progenies.

## Conclusion

Different publications on sunflower and lettuce genetic transformation were reviewed in this work, and it is concluded that these *Asteraceae* species have certain specific characteristics of their own.

In the case of sunflower, despite the importance of this crop, the delay in its biotechnological improvement could be due to two main reasons: its poor response to tissue culture and its very low regeneration levels, for which it is still considered a recalcitrant species, and the extensive use of the *CaMV35S* promoter. We believe that the application of regeneration-promoting genes and the use of more suitable promoters will allow the obtaining of biotechnologically improved sunflower plants stably expressing the traits of interest through generations.

In lettuce, a highly responsive species to tissue culture, it is possible to regenerate and obtain fertile plants from numerous explants, even by protoplast regeneration. Moreover, nuclear and chloroplast genetic transformation has been successfully implemented to improve crop characteristics or to produce molecules of interest. This plasticity enabled the rapid implementation of CRISPR-mediated gene editing protocols. As in sunflower, it is advisable to avoid the use of the *CaMV35S* promoter in genetic transformation.

Although *Arabidopsis* and tobacco are commonly used as model species, evidence shows that genetic constructs that work successfully in these species may present difficulties in the *Asteraceae* species mentioned in this review. This situation indicates the advantage of using lettuce as a model plant, as was previously discussed. The advancement of sunflower and lettuce biotechnological improvement will also depend on the search and implementation of appropriate regulatory elements for these species.

It will be possible to continue advancing in the genetic transformation and gene editing of these plant species taking into account all these considerations.

## Author Contributions

FD, LR, and MLB contributed equally to this manuscript, including writing and discussion. VB participated in the discussion. HH revised the manuscript. All authors read and approved the final manuscript.

## Funding

This work was supported by IABIMO UEDD INTA-CONICET and by INTA (Instituto Nacional de Tecnología Agropecuaria), Argentina, grant numbers PNHFA I508 and PNBIO I115. FD is a postdoctoral fellow of INTA-CONICET (Instituto Nacional de Tecnología Agropecuaria - Consejo Nacional de Investigaciones Científicas y Técnicas).

## Conflict of Interest

The authors declare that the research was conducted in the absence of any commercial or financial relationships that could be construed as a potential conflict of interest.

## Publisher’s Note

All claims expressed in this article are solely those of the authors and do not necessarily represent those of their affiliated organizations, or those of the publisher, the editors and the reviewers. Any product that may be evaluated in this article, or claim that may be made by its manufacturer, is not guaranteed or endorsed by the publisher.
